# Efficacy of conservative intervention for kinesiophobia in individuals with a history of ankle sprain: A systematic review

**DOI:** 10.1002/pmrj.13328

**Published:** 2025-02-19

**Authors:** Takumi Kobayashi, Yuta Koshino

**Affiliations:** ^1^ Department of Rehabilitation Sciences Graduate School of Health Sciences, Gunma University Maebashi Japan; ^2^ Faculty of Health Sciences, Hokkaido University Sapporo Japan

## Abstract

**Objective:**

To determine the effect of conservative interventions on kinesiophobia, a fear‐avoidance belief regarding exercise, in individuals who have had a lateral ankle sprain.

**Literature Survey:**

Systematic computerized literature search was performed using PubMed, CINAHL, Web of Sciences, and Cochrane Library databases. Randomized controlled trials investigating the effects of conservative interventions on kinesiophobia in individuals with a history of lateral ankle sprain, including chronic ankle instability, compared to those of control, sham, or different conservative interventions were included. They were written in English and published prior to December 2023.

**Methodology:**

Two independent reviewers screened the studies using specific eligibility criteria. Study characteristics, patients, intervention and comparator, and outcome were extracted. Outcomes were defined as kinesiophobia observed using a questionnaire (eg, Tampa Scale for Kinesiophobia‐11) or other means. The risk of bias was assessed using the Revised Cochrane risk of bias tool for randomized trials.

**Synthesis:**

Five randomized controlled trials were included. These studies examined the effects of transcranial direct current stimulation, joint mobilization, balance and strength training, visual biofeedback during multimodal training, and low‐friction patches attached to the outside of shoes, respectively. Limited evidence from single studies showed that the visual biofeedback during walking and low‐friction patches attached on the outside of shoes were significantly more effective than the control and other treatments for kinesiophobia in individuals with a history of a lateral ankle sprain. Because the intervention and control groups differed between studies, data synthesis through meta‐analysis was not performed.

**Conclusions:**

Visual biofeedback during walking and low‐friction patches attached on the outside of shoes may improve kinesiophobia in patients with a history of lateral ankle sprains. An approach to sensory‐perceptual impairment in individuals with a history of lateral ankle sprains may be more effective in improving kinesiophobia. These conservative interventions may have an effect on kinesiophobia, but the evidence is limited.

## INTRODUCTION

Ankle sprain is one of the most common lower‐extremity injuries and has a high recurrence rate. Recurrent ankle sprains can lead to chronic ankle instability (CAI). CAI results in various structural and functional joint impairments, leading to a decline in health‐related quality of life^1^ and increases the risk of developing ankle osteoarthritis in the future.^2^ Therefore, there is a need to consider efficient therapies for CAI.

CAI includes a variety of pathological conditions, including “pathomechanical,” “motor‐behavioral,” and “sensory‐perceptual” impairments^3^ and several previous studies have reported pathomechanical^4^ and motor‐behavioral abnormalities^5^, ^6^ in CAI. However, recently published clinical guidelines on ankle sprains and CAI now recommend evaluation of psychological factors, such as kinesiophobia,^7^ and several studies have shown that fear of injury in individuals with CAI may prevent a return to sports.^1^, ^8^ Recognizing kinesiophobia is important for facilitating the return to sports, as fear of reinjury can affect recovery as a barrier to return to sports.^9^, ^10^ Several studies have shown that kinesiophobia is higher in college athletes or physically active participants with a history of ankle sprain or CAI than in healthy controls.^11^, ^12^, ^13^ Therefore, improving psychological factors is one of the important aspects to be considered in athletes with CAI who wish to return to sports.

CAI is often treated conservatively using manual therapy, strength training, and exercise training.^7^, ^14^ The aim of conservative treatment is to correct modifiable deficits, such as reduced muscle strength, decreased neuromuscular control, impaired proprioception, and altered gait patterns that are observed in individuals with CAI.^15^ In CAI, various conservative interventions have been shown to improve balance ability, neuromuscular control, and motor patterns, as well as prevent recurrent ankle sprains.^16^, ^17^ However, the optimal conservative treatment for patients with previous ankle sprains, including CAI, has not been clarified, and to our knowledge, it is currently unknown whether conservative interventions can improve kinesiophobia in patients with CAI.

This systematic review aimed to investigate the effect of conservative interventions on kinesiophobia in individuals with a history of a lateral ankle sprain. The findings may contribute to the development of optimal conservative treatments for patients with repeated ankle sprains, including CAI, whose return to sports is delayed because of kinesiophobia.

## METHODOLOGY

This study was conducted in accordance with the Preferred Reporting Items for Systematic Reviews and Meta‐Analysis guidelines for reporting systematic reviews.^18^ The protocol for this systematic review was prospectively registered with PROSPERO (CRD 42022301902).

### Search strategy

PubMed, CINAHL, Web of Sciences, and Cochrane Library databases were searched from inception to March 1, 2022 and searched again on December 5, 2023. The search strategy for each database is presented in Appendix S1. The search results were exported to Endnote X9 (Thomson Reuters, New York, USA). The reference lists of the relevant systematic reviews were also screened manually. Two independent reviewers (T.K., Y.K.) screened the titles and abstracts of the studies after excluding duplicates. Screening discrepancies between the reviewers were discussed and resolved. Reviewers independently screened the full texts of the remaining studies using specific eligibility criteria. Disagreements during screening were resolved by consensus between the two reviewers.

### Eligibility criteria

We included randomized controlled trials (RCTs) in English language and studies that met the following criteria.

#### Patients

Patients with lateral ankle sprains and CAI, including functional and mechanical instability, were included. According to the criteria of the International Ankle Consortium,^19^ CAI is defined as a history of multiple ankle sprains and symptoms such as pain and instability. Patients with acute ankle sprains were excluded from the study.

#### Intervention and comparator

Patients who received conservative treatment were included, and those who received operative and pharmacotherapy treatments were excluded. Control conservative treatment, sham, wait‐and‐see, and no treatment groups were included as comparators.

#### Outcome

Studies that measured kinesiophobia outcomes were also included. Kinesiophobia outcomes were defined as the kinesiophobia observed by the questionnaire or other means.

### Data extraction

Two reviewers (T.K., Y.K.) independently extracted the following data, and any disagreements were resolved by consensus between the two reviewers.

#### Study characteristics

Authors' names and year of publication.

#### Patients

Total sample size, mean age, and inclusion criteria.

#### Intervention and comparator

Intervention type, frequency, and duration. When multiple comparator groups existed, the values of the groups with some interventions were combined into one group and compared with those of the control group.^20^

#### Outcome

Means and SDs of the kinesiophobia outcome measured at the follow‐up time point closest to the end of the intervention period were extracted. In the present study, patients reported assessments such as the Tampa Scale for Kinesiophobia‐11 (TSK‐11)^21^ and the Fear‐Avoidance Beliefs Questionnaire (FABQ),^22^ which have been validated in previous studies, or participants' quantified fear of exercise or injury, including the visual analog scale among others, as a kinesiophobia outcome. If multiple outcomes were reported for kinesiophobia outcomes, the most frequently adopted outcome in the selected studies was recruited to reduce heterogeneity.^23^ One study reported the median and quartiles, so the Box–Cox method was used to estimate the means and SDs.^23^ If the outcome data were not provided in the included papers, we emailed the corresponding author to request the data. Studies with data presented in the graph but without a response to the data request were extracted using WebPlotDigitizer (https://automeris.io/WebPlotDigitizer).^24^

### Risk of bias assessment

Two reviewers independently assessed the included studies using the revised Cochrane risk of bias tool for randomized trials.^25^ This tool consists of the randomization process, intended interventions, missing outcome data, measurement of the outcome, and selection of the reported result. The risk (low, some concerns, high) of each domain was determined by the evaluator answering the signaling question for each domain with “Yes,” “Probably yes,” “Probably no,” “No,” and “No information.” Disagreements in the risk of bias assessment were resolved by consensus between the two reviewers. If any of the domains were at high risk, the study was considered high risk; if all the domains were at low risk, it was considered low risk. If there were some concerns in at least one domain but no high risk of bias in any of the domains, the study was considered to have some concerns.

### Data synthesis

Review Manager 5.4.1 (RevMan; The Cochrane Collaboration, 2020) was used for all data analyses and syntheses. Data from the same intervention categories were pooled for the kinesiophobia outcome measures. Standardized mean differences (SMD) with 95% confidence intervals (CIs) were calculated from the data at the end of the intervention in each study. The direction of the kinesiophobia variable was corrected to pool the data and calculate SMD. A positive SMD indicated that the intervention improved the kinesiophobia outcome when compared to comparators. For interventions that showed a positive effect, we checked whether the minimal clinically important difference had been calculated and whether the change was clinically meaningful. Meta‐analysis (random effects) was performed to compare each conservative intervention with the control, sham, and other intervention groups. We interpreted a difference as statistically significant when the 95% CI of the pooled SMD did not reach 0. The pooled SMD was interpreted as follows: <0.40, small effect; 0.40–0.70, moderate effect; and >0.70, large effect.^20^ Statistical heterogeneity was assessed using the I^2^ statistic as follows: 0%–40%, might not be important; 30%–60%, moderate heterogeneity; 50%–90%, substantial heterogeneity; 75%–100%, considerable heterogeneity.^20^

## RESULTS

### Study selection and characteristics

**FIGURE 1:**
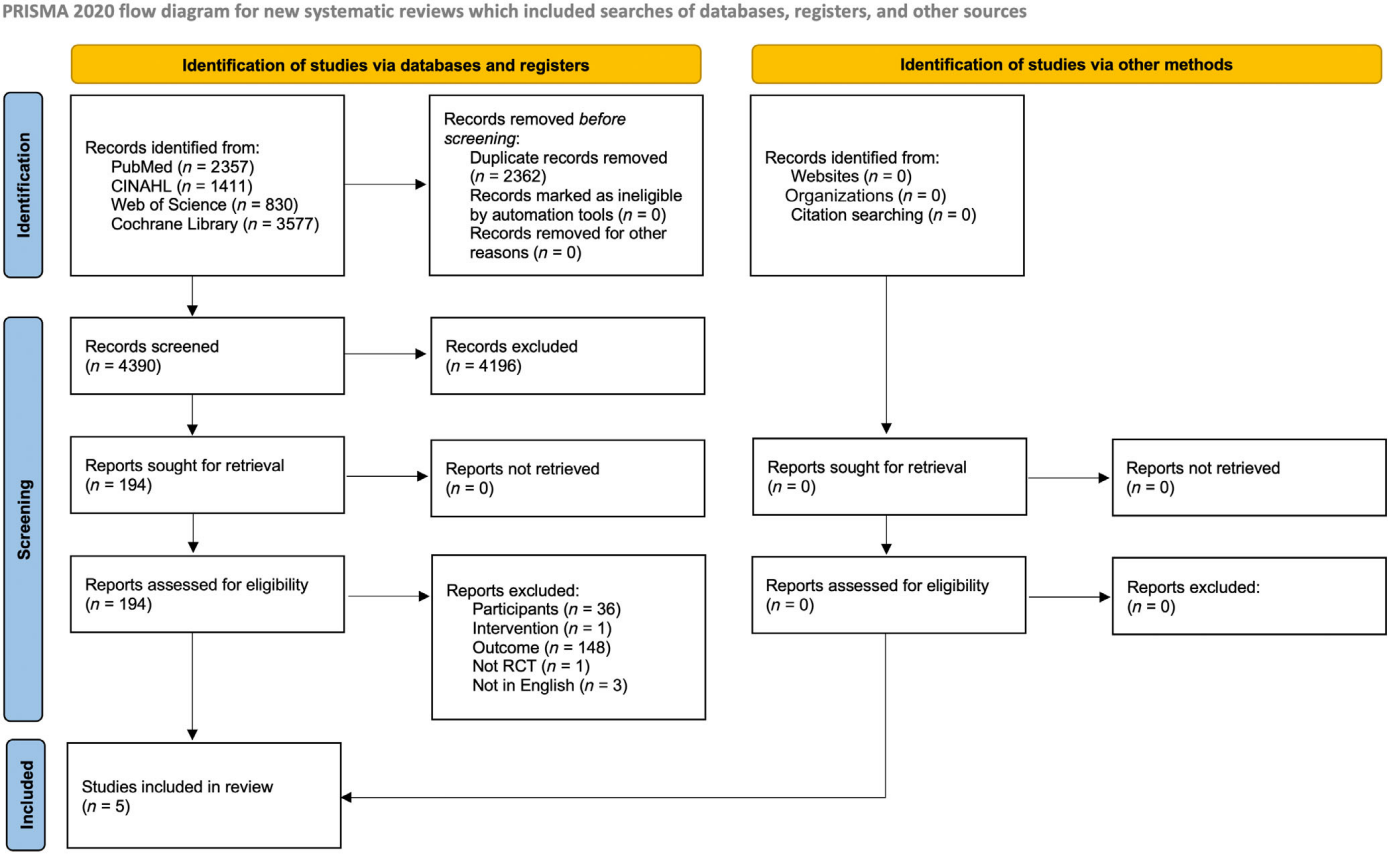
Preferred Reporting Items for Systematic Reviews and Meta‐Analysis (PRISMA) 2020 flow diagram. RCT, randomized controlled trial.

The flow and results of the study selection process are shown in Figure 1. After the screening process, 616 patients from five RCTs that met the selection criteria were included in this systematic review (Table 1). The mean age of the study participants ranged from 20.6 to 24.8 years. Kinesiophobia outcomes were TSK‐11 (*n* = 3),^23^, ^26^, ^27^ FABQ (*n* = 2),^23^, ^24^ and fear of injury (*n* = 1)^28^; only one study employed two outcomes (TSK‐11 and FABQ).^23^

**TABLE 1:** Study characteristics.

Authors	No. Total	Age (years)	Inclusion criteria for participants	Intervention	Intervention frequency and duration	Kinesiophobia outcome
Bruce et al., 2020^26^	26	G1: 22.2 (2.8) G2: 22.5 (3.2)	(1) an AS more than 1 year before; (2) recurrent sensations of rolling or GW; (3) IdFAI score >10.	G1: **TDCS** **±** **strengthening** (anodal TDCS with eccentric ankle strength training) G2: **Strength training** (sham and eccentric ankle strength training)	Total 10 times 4 weeks	TSK‐11
Burton et al., 2020^23^	18	G1: 20.6 (2.07) G2: 21.0 (2.2)	(1) at least one AS; (2) at least two episodes of GW in the last 3 months; (3) AII score of 4 or more, and CAIT score 24 or less; (4) Godin Leisure‐Time Exercise Questionnaire score 24 or more.	G1: **Joint mobilization** (clinician‐applied talocrural joint mobilization) G2: **Self‐joint mobilization** (self‐applied talocrual joint mobilization)	Total 6 times 2 weeks	TSK‐11, FABQ
Hall et al., 2018^24^	47	G1: 21.5 (3.8) G2: 21.3 (3.1) G3: 19.9 (4.5)	(1) at least one AS; (2) multiple episodes of the ankle GW, recurrent sprain, and “feelings of instability” in the 6 months before; (3) IdFAI score of 11 or more.	G1: **Balance training** (progressive hop‐to‐stabilization balance training) G2: **Strength training** (proprioceptive neuromuscular facilitation and heel raises) G3: **Control** (bicycle)	3 times/week 6 weeks	FABQ
Koldenhoven et al., 2021^27^	43	G1: 22.23 (3.8) G2: 21.5 (3.0)	(1) AS at least 12 months before; (2) FAAM Sport score of 85% or less; (3) feelings of perceived instability (IdFAI score > 10).	G1: **Multimodal** **±** **biofeedback** (impairment‐based progressive rehabilitation and visual biofeedback to reduce ankle inversion during treadmill walking) G2: **Multimodal** (impairment‐based progressive rehabilitation)	Total 8 times 4 weeks	TSK‐11
Lysdal et al., 2021^28^	510	G1: 22.3 (4.0) G2: 23.0 (4.5)	(1) at least one LAS in the preceding 24 months.	G1: **Spraino** (low‐friction patches attached on the outside of indoor sports shoes) G2: **Control** (usual indoor sports shoes)	9 months	Fear of injury

*Note*: Bold and underlined texts indicate the extracted groups and outcomes.

Abbreviations: AII, Ankle Instability Instrument; AS, ankle sprain; CAIT, Cumberland Ankle Instability Tool; FAAM, Foot and Ankle Ability Measure; FABQ, Fear‐Avoidance Beliefs Questionnaire; G, group; GW, giving way; IdFAI, Identification of Functional Ankle Instability; LAS, lateral ankle sprain; TDCS, transcranial direct current stimulation; TSK‐11, Tampa Scale for Kinesiophobia‐11.

### Risk of bias

**FIGURE 2:**
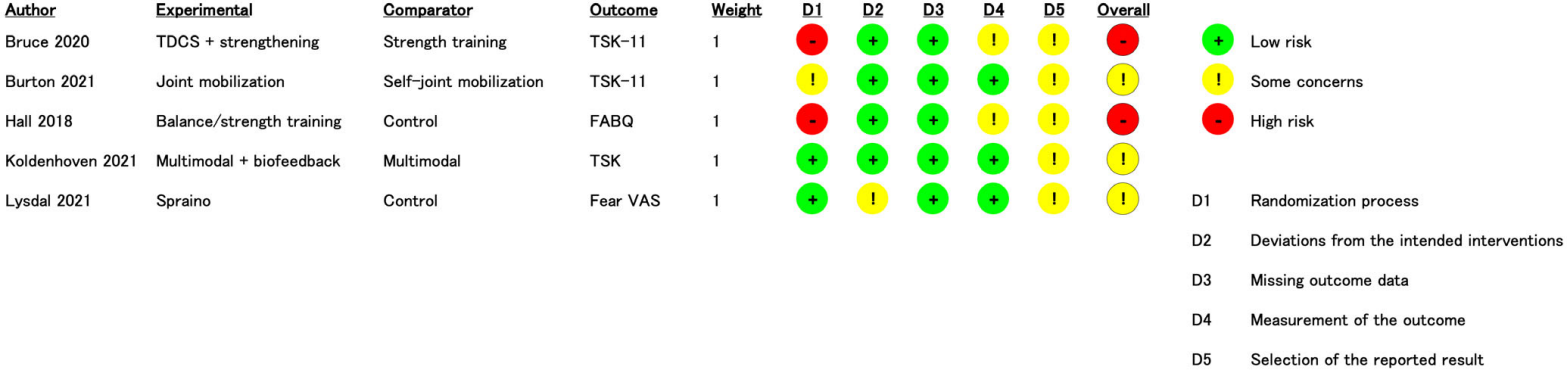
The risk of bias assessment of the included studies. FABQ, Fear‐Avoidance Beliefs Questionnaire; TDCS, transcranial direct current stimulation; TSK‐11, Tampa Scale for Kinesiophobia‐11; VAS, visual analog scale.

The results and summary of the bias risk assessments are shown in Figure 2. Three studies were considered to have “some concerns,” and two were considered “high risk.” The two studies that were considered high risk were at risk during the randomization process. All studies were considered to have “some concerns” in the selection of the reported results.

### Effects on kinesiophobia outcome

**FIGURE 3:**
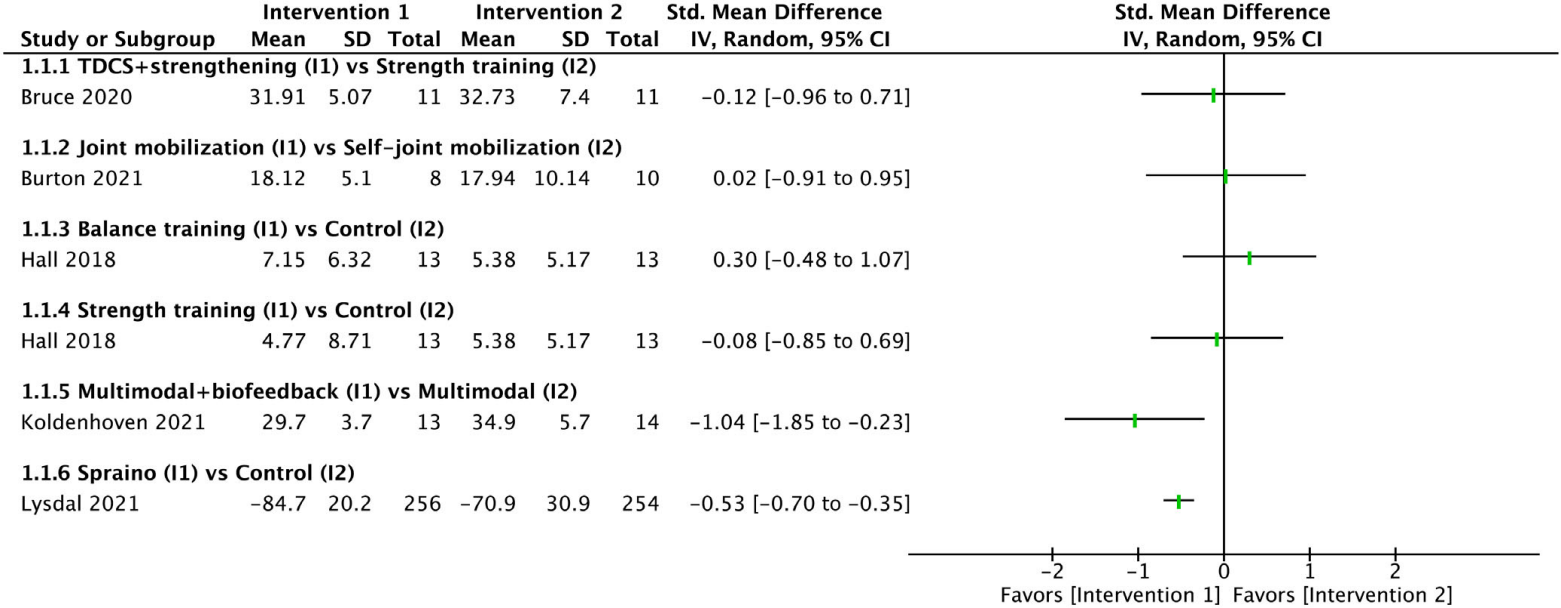
Summary of the effects of conservative interventions on kinesiophobia. I1, intervention 1; I2, intervention 2; CI, confidence interval; IV, inverse variance; TDCS, transcranial direct current stimulation.

Because all studies had different interventions and control groups, data synthesis through meta‐analysis was not performed. The results of included studies is showed in Figure 3. Limited evidence from single studies showed that the visual biofeedback to reduce ankle inversion during treadmill walking and low‐friction patches attached on the outside of shoes had a better effect on kinesiophobia outcome than control group. The other interventions, which were anodal transcranial direct current stimulation, clinician‐applied talocrural joint mobilization, balance training, and strength training, had no significant effect. Because the intervention and control groups differed between studies, the assessment of the certainty of the evidence was not applicable.

## DISCUSSION

This systematic review aimed to investigate the effectiveness of conservative interventions in improving kinesiophobia outcomes in individuals with a history of lateral ankle sprains. The visual biofeedback during walking and low‐friction patches attached on the outside of shoes significantly improved kinesiophobia outcomes in individuals with a history of lateral ankle sprains. However, this evidence was limited to the results of single studies.

Kinesiophobia factors are associated with many musculoskeletal disorders, such as anterior cruciate ligament injury^29^ and low back pain.^30^ Improving kinesiophobia outcomes is an important component in treating patients with musculoskeletal disorders.^31^ In CAI, subjective instability is considered to be one of the most important pathophysiological factors, as well as structural and functional instability, and evaluation of kinesiophobia outcomes is recommended in patients with ankle sprains.^3^, ^7^ Previous studies have shown that physically active individuals from a university community with a history of lateral ankle sprain and physically active participants with CAI have higher fear avoidance levels compared to the fear levels of controls.^13^ Another study on college athletes reported that functional ankle instability was associated with fear of movement or reinjury.^11^ In addition, Watanabe et al.^32^ showed that higher kinesiophobia is associated with higher perceived instability in athletes with CAI. Therefore, the evaluation of kinesiophobia outcomes is an important factor in the treatment of patients with a history of lateral ankle sprain or CAI. Meanwhile, the contribution of psychological factors, including kinesiophobia, to impaired recovery in patients with CAI has not been clarified; this is considered an important future research topic.

Questionnaires assessing kinesiophobia aspects, such as fear of movement, include TSK‐11,^21^ FABQ,^22^ and pain catastrophizing scale.^33^ The TSK‐11^23^, ^26^, ^27^ and FABQ^23^, ^24^ were also used in the studies included in this systematic review, and showed a significant effect of some conservative intervention. Five RCTs were included in this systematic review, and all examined the effects of different interventions and intervention durations. One study examined the effects of a 2‐week intervention of clinician‐applied mobilizations, primarily an approach to pathomechanical impairments in CAI.^23^ Another study took a 6‐week approach to balance deficits and muscle weakness in motor‐behavioral impairments.^24^ Two studies examined the effects of anodal transcranial direct current stimulation added to strength training^26^ and visual feedback during gait training.^27^ The main approach of these studies was to address sensory‐perceptual impairments. In another study, Lysdal et al.^28^ examined the effects of a 9‐month intervention using low‐friction patches applied to the outside of the shoes.

Of these five RCTs, two individual studies with interventions using visual feedback and low‐friction patches showed that the intervention had significant effects on kinesiophobia in individuals with a history of lateral ankle sprains (Figure 3).^27^, ^28^ Although the mechanisms by which these individual interventions improve kinesiophobia outcomes are not fully understood, the approach to sensory‐perceptual impairments in individuals with a history of lateral ankle sprain may be more effective in improving kinesiophobia.^3^ However, the types of interventions that improve kinesiophobia outcomes in individuals with a history of lateral ankle sprain are currently unknown, and further RCTs are needed.

The clinical implications of our findings are that visual biofeedback during walking for up to 20 minutes on a total of 8 days over 4 weeks and low‐friction patches attached to the outside of shoes during a 9‐month intervention in patients with a history of lateral ankle sprain may improve kinesiophobia. However, the evidence is limited and should be interpreted with caution, as the improvement in kinesiophobia by conservative interventions may differ between individuals. It is also difficult to identify the most effective types of interventions for improving kinesiophobia. Therefore, further validation using RCTs is required.

### Study limitations

This systematic review has four limitations. First, none of the studies included in this review had a low risk of bias. This is mainly due to the measurement of the outcome and selection of the reported results, which may have been due to the impracticality of blinding for interventions, such as exercise therapy and manual therapy, as well as the need for more agreement between the results determined in the prior protocol and the results in the article. Therefore, the results of this study should be interpreted with caution. Second, we found diversity in the type and duration of interventions across studies; therefore we were not able to examine differences in effects by intervention duration. Moreover, because minimal clinically important differences in populations with previous ankle sprain or CAI have not been reported, it is not clear whether the changes are clinically meaningful in interventions that have been found to be effective. Finally, this review included only articles published in English, which might have increased the risk of publication bias.

## CONCLUSIONS

This review revealed that there is limited evidence for successful treatments of kinesiophobia in patients with a history of lateral ankle sprain. However, the available evidence from individual studies suggests that visual biofeedback during walking and low‐friction patches attached on the outside of shoes are effective in improving kinesiophobia. Improving sensory‐perceptual impairments in individuals with a history of lateral ankle sprains may be effective in improving kinesiophobia. However, the risk of bias was moderate to high for each study, and the type and duration of intervention varied across studies. Further RCTs are needed to determine the most effective interventions for improving kinesiophobia in patients with a history of lateral ankle sprain.

## FUNDING INFORMATION

This study was funding supported by the Grants‐in‐Aid for Scientific Research of Japan Society for the Promotion of Science, Grant Number 23K10511.

## DISCLOSURES

The autors declare no potential conflict of interest associated with this manuscript.

This journal‐based CME activity is designated for 1.0 *AMA PRA Category 1 Credit* ^TM^. Effective January 2024, learners are no longer required to correctly answer a multiple‐choice question to receive CME credit. Completion of an evaluation is required, which can be accessed using this link, https://onlinelearning.aapmr.org/. This activity is FREE to AAPM&R members and available to nonmembers for a nominal fee. CME is available for 3 years after publication date. For assistance with claiming CME for this activity, please contact (847) 737–6000. All financial disclosures and CME information related to this article can be found on the Online Learning Portal (https://onlinelearning.aapmr.org/) prior to accessing the activity.

## Supporting information


**Appendix S1:** Search strategies.
