# Supplementary material for: Efficacy of conservative intervention for kinesiophobia in individuals with a history of ankle sprain: A systematic review
Source: PM R. 2025 Feb 19;17(7):822–9. doi: 10.1002/pmrj.13328 (PMC12238745; doi:10.1002/pmrj.13328)
Supplement: Supplementary file 1 — Appendix S1: Search strategies. [file PMRJ-17-822-s001.docx]

**Appendix A.** Search strategies

**PubMed**

| No | **Query** |
| --- | --- |
| #1 | "ankle"[all] OR "talocrural"[all] |
| #2 | "ankle"[mh] OR "ankle joint"[mh] OR "lateral ligament, ankle"[mh] |
| #3 | #1 OR #2 |
| #4 | "sprain"[all] OR "injury"[all] OR "instability"[all] OR "ankle instability"[all] OR "recurrent[all]" OR "repetitive"[all] OR "repeated"[all] OR "chronic"[all] |
| #5 | "ankle Injuries"[mh] OR "joint instability"[mh] OR "sprains and strains"[mh] |
| #6 | #4 OR #5 |
| #7 | #3 AND #6 |
| #8 | "conservative treatment"[all] OR "nonoperative treatment"[all] OR "nonsurgical treatment"[all] OR "rehabilitation"[all] OR "physical therapy"[all] OR physiotherapy[all] OR "training"[all] OR "balance training"[all] OR "neuromuscular training"[all] OR "proprioceptive training"[all] OR "strength training"[all] OR "plyometric training"[all] OR "exercise therapy"[all] OR "wobble board"[all] OR "massage"[all] OR "stretching"[all] OR "manual therapy"[all] OR "manipulation"[all] OR "joint mobilization"[all] OR "orthotics"[all] OR "orthosis"[all] OR "foot wear"[all] OR "taping"[all] OR "cryotherapy"[all] OR "ultrasound therapy"[all] OR "electrical therapy"[all] OR "electrotherapy"[all] OR "vibration"[all] OR "complementary therapy"[all] OR "physical agents"[all] OR "physical modalities"[all] |
| #9 | "conservative treatment"[mh] OR "rehabilitation"[mh] OR "exercise therapy"[mh] OR "muscle stretching exercises"[mh] OR "musculoskeletal manipulations"[mh] OR "cryotherapy"[mh] OR "patient education as topic"[mh] OR "physical therapy specialty"[mh] OR "complementary therapies"[mh] OR "athletic tape"[mh] OR "foot orthoses"[mh] OR "shoes"[mh] OR "orthotic devices"[mh] |
| #10 | #8 OR #9 |
| #11 | "clinical trial"[all] OR "controlled trial"[all] OR "random"[all] OR "comparative study" [all] OR "crossover trial"[all] |
| #12 | "Randomized Controlled Trial"[pt] OR "clinical trial"[pt] OR "comparative study"[pt] |
| #13 | #11 OR #12 |
| #14 | #7 AND #10 AND #13 |

**Cochrane Library**

| No | **Query** |
| --- | --- |
| #1 | "ankle" OR "talocrural" |
| #2 | [mh "ankle"] OR [mh "ankle joint"] OR [mh "lateral ligament, ankle"] |
| #3 | #1 OR #2 |
| #4 | "sprain" OR "injury" OR "instability" OR "ankle instability" OR "recurrent" OR "repetitive" OR "repeated" OR "chronic" |
| #5 | [mh "ankle Injuries"] OR [mh "joint instability"] OR [mh "sprains and strains"] |
| #6 | #4 OR #5 |
| #7 | #3 AND #6 |
| #8 | "conservative treatment" OR "nonoperative treatment" OR "nonsurgical treatment" OR "rehabilitation" OR "physical therapy" OR "physiotherapy" OR "training" OR "balance training" OR "neuromuscular training" OR "proprioceptive training" OR "strength training" OR "plyometric training" OR "exercise therapy" OR "wobble board" OR "massage" OR "stretching" OR "manual therapy" OR "manipulation" OR "joint mobilization" OR "orthotics" OR "orthosis" OR "foot wear" OR "taping" OR "cryotherapy" OR "ultrasound therapy" OR "electrical therapy" OR "electrotherapy" OR "vibration" OR "complementary therapy" OR "physical agents" OR "physical modalities" |
| #9 | [mh "conservative treatment"] OR [mh "rehabilitation"] OR [mh "exercise therapy"] OR [mh "muscle stretching exercises"] OR [mh "musculoskeletal manipulations"] OR [mh "cryotherapy"] OR [mh "patient education as topic"] OR [mh "physical therapy specialty"] OR [mh "complementary therapies"] OR [mh "athletic tape"] OR [mh "foot orthoses"] OR [mh "shoes"] OR [mh "orthotic devices"] |
| #10 | #8 OR #9 |
| #11 | "clinical trial" OR "controlled trial" OR "random" OR "comparative study" OR "crossover trial" |
| #12 | "Randomized Controlled Trial":pt OR "clinical trial":pt |
| #13 | #11 OR #12 |
| #14 | #7 AND #10 AND #13 |

**Web of Sciences**

| No | **Query** |
| --- | --- |
| #1 | TS="ankle" OR TS="talocrural" |
| #2 | TS="sprain" OR TS="injury" OR TS="instability" OR TS="ankle instability" OR TS="recurrent" OR TS="repetitive" OR TS="repeated" OR TS="chronic" |
| #3 | #1 AND #2 |
| #4 | TS="conservative treatment" OR TS="nonoperative treatment" OR TS="nonsurgical treatment" OR TS="rehabilitation" OR TS="physical therapy" OR TS="physiotherapy" OR TS="training" OR TS="balance training" OR TS="neuromuscular training" OR TS="proprioceptive training" OR TS="strength training" OR TS="plyometric training" OR TS="exercise therapy" OR TS="wobble board" OR TS="massage" OR TS="stretching" OR TS="manual therapy" OR TS="manipulation" OR TS="joint mobilization" OR TS="orthotics" OR TS="orthosis" OR TS="foot wear" OR TS="taping" OR TS="cryotherapy" OR TS="ultrasound therapy" OR TS="electrical therapy" OR TS="electrotherapy" OR TS="vibration" OR TS="complementary therapy" OR TS="physical agents" OR TS="physical modalities" |
| #5 | TS="clinical trial" OR TS="controlled trial" OR TS="random" OR TS="comparative study" OR TS="crossover trial" |
| #6 | #3 AND #4 AND #5 |

**CINAHL**

| No | **Query** |
| --- | --- |
| #1 | TX "ankle" OR TX "talocrural" |
| #2 | MH "ankle" OR MH "ankle joint" OR MH "lateral ligament, ankle" |
| #3 | S1 OR S2 |
| #4 | TX "sprain" OR TX "injury" OR TX "instability" OR TX "ankle instability" OR TX "recurrent" OR TX "repetitive" OR TX "repeated" OR TX "chronic" |
| #5 | MH "ankle Injuries" OR MH "joint instability" OR MH "sprains and strains" |
| #6 | S4 OR S5 |
| #7 | S3 AND S6 |
| #8 | TX "conservative treatment" OR TX "nonoperative treatment" OR TX "nonsurgical treatment" OR TX "rehabilitation" OR TX "physical therapy" OR TX "physiotherapy" OR TX "training" OR TX "balance training" OR TX "neuromuscular training" OR TX "proprioceptive training" OR TX "strength training" OR TX "plyometric training" OR TX "exercise therapy" OR TX "wobble board" OR TX "massage" OR TX "stretching" OR TX "manual therapy" OR TX "manipulation" OR TX "joint mobilization" OR TX "orthotics" OR TX "orthosis" OR TX "foot wear" OR TX "taping" OR TX "cryotherapy" OR TX "ultrasound therapy" OR TX "electrical therapy" OR TX "electrotherapy" OR TX "vibration" OR TX "complementary therapy" OR TX "physical agents" OR TX "physical modalities" |
| #9 | MH "rehabilitation" OR MH "athletic training" OR MH "physical therapy" OR MH "manual therapy" OR MH "joint mobilization" OR MH "cryotherapy" OR MH "patient education" OR MH "physical therapy practice, research-based" OR MH "alternative therapies" OR MH "orthoses" OR MH "athletic tape" OR MH "foot orthoses" OR MH "shoes" |
| #10 | S8 OR S9 |
| #11 | TX "clinical trial" OR TX "controlled trial" OR TX "random" OR TX "comparative study" OR TX "crossover trial" |
| #12 | PT "randomized controlled trial" OR PT "clinical trial" |
| #13 | S11 OR S12 |
| #14 | S7 AND S10 AND S13 |
